# Occupational health risks from climate-related and infectious-disease stressors: A scoping review of worker safety and resilience

**DOI:** 10.1371/journal.pone.0361296

**Published:** 2026-10-21

**Authors:** Muhammed Abdul Odud, Omar I. Abdul-Aziz

**Affiliations:** 1 Department of Industrial and Management Systems Engineering (IMSE), Benjamin M. Statler College of Engineering and Mineral Resources, West Virginia University, Morgantown, West Virginia, United States of America; 2 Wadsworth Department of Civil and Environmental Engineering, Benjamin M. Statler College of Engineering and Mineral Resources, West Virginia University, Morgantown, West Virginia, United States of America; Università degli Studi di Milano: Universita degli Studi di Milano, ITALY

## Abstract

**Objective:**

This scoping review examines how climate-related stressors and infectious-disease conditions, including pandemic-related workplace disruptions, interact to influence worker health, vulnerability, and occupational resilience. The review focuses on shared exposure pathways, vulnerable worker populations, methodological patterns, policy gaps, and strategies for strengthening worker safety under compound hazards. Rather than treating pandemics as isolated events, the review uses pandemic conditions as a lens to understand broader, recurring occupational risks and to inform strategies for building safer and more resilient workplaces under compound hazards.

**Methods:**

Following PRISMA-ScR guidelines, a structured literature search was conducted in PubMed, Scopus, Web of Science, and Google Scholar for English-language peer-reviewed and formally structured evidence sources published primarily between January 2020 and April 2025, with one pre-2020 policy study retained for foundational context. Out of 612 records initially identified, 10 studies or sources met the inclusion criteria across healthcare, agriculture, construction, emergency response, and other high-exposure sectors after systematic screening and eligibility assessment. Data were charted and synthesized by occupational sector, geographic region, exposure type, worker vulnerability, and methodological approach.

**Results:**

The evidence indicates that climate-related and infectious-disease stressors frequently converge, amplifying physical, environmental, and psychosocial pressures on workers. Disproportionate risks were observed among migrant workers, informal laborers, low-income populations, older adults, women, and seasonal or cross-border agricultural workers, particularly where access to healthcare, legal protections, or adaptive infrastructure was limited. Most studies relied on descriptive, qualitative, cross-sectional, conceptual, or review-based designs, with limited use of predictive modeling, geospatial analysis, longitudinal evidence, or integrated datasets. Policy responses were often fragmented, with climate adaptation and infection-control measures addressed separately despite overlapping benefits.

**Conclusions:**

Climate-related and infectious-disease stressors interact to reinforce occupational health inequities and expose persistent gaps in worker protection. The findings highlight the need for integrated, multi-hazard occupational health strategies that align climate adaptation, infection prevention, and psychosocial support. Embedding data-driven early-warning systems, equitable protections for vulnerable workers, and cross-sector coordination into occupational policies may help strengthen workforce resilience during routine operations as well as future climate extremes and public health crises. However, the findings should be interpreted with caution because of the limited number of available studies combining climate-related and infectious-disease occupational risks, heterogeneous study designs, and the emerging nature of the evidence base. Furthermore, since this scoping review maps evidence rather than formally grading evidence strength or testing causal relationships, recommendations should be interpreted as evidence-informed directions for future occupational-health planning and research.

## 1. Introduction

Worker health is increasingly shaped by two overlapping global challenges: pandemics and climate change. Each poses significant risks independently; however, when they occur together, they generate compounding hazards that intensify exposure and strain existing workplace protections. Workers in agriculture, construction, transportation, and delivery services often face extreme heat and degraded air quality while simultaneously adhering to infection-prevention measures. Similarly, healthcare providers, firefighters, and paramedics frequently experience long shifts, elevated infection risk, and sustained psychosocial stress. These overlapping pressures disproportionately affect workers with fewer protections, including migrant workers, part-time employees, informal laborers, and seasonal agricultural workers [1–4].

Although research examining pandemic-related and climate-related impacts on worker health has expanded, most studies continue to address these hazards separately. This separation limits understanding of how environmental, biological, and social stressors interact in real-world occupational settings. For example, protective clothing intended to reduce infection transmission may increase thermal strain for outdoor and high-exertion workers, while inadequate indoor ventilation can simultaneously elevate heat exposure and airborne-pathogen risk. These dynamics underscore the need for integrated, multi-hazard workplace-safety strategies rather than siloed approaches focused on single threats [5–7]. Fragmented policy responses, in which climate-adaptation measures and infection-control guidance operate independently, further complicate compliance and weaken protection for high-risk worker populations.

This review applies the PRISMA-ScR (Preferred Reporting Items for Systematic Reviews and Meta-Analyses Extension for Scoping Reviews) framework to map and synthesize the available evidence on how climate-related and infectious-disease stressors intersect within occupational-health contexts [8]. Rather than testing formal hypotheses, the review is guided by two analytical propositions: (1) occupational-health risks are shaped by social and economic factors, including income, job characteristics, employment conditions, and access to healthcare; and (2) worker protection is influenced by interconnected determinants operating at the individual, workplace, community, and policy levels [9,10].

This review addresses a critical evidence gap by jointly examining pandemic-related and climate-related stressors within occupational-health contexts—a relationship that remains underexplored within systematic and scoping review frameworks. Guided by this conceptual framing, the review addresses five key questions:

(1) What compounding hazards arise when pandemic-related and climatic stressors occur together?
(2) Which worker populations are most affected by these combined risks?
(3) How are these intersecting risks currently studied?
(4) Where do gaps in protection and policy persist? and
(5) What actions could strengthen occupational resilience during future crises?

## 2. Materials and methods

### 2.1. Search strategy and information sources

This scoping review was conducted in accordance with the Preferred Reporting Items for Systematic Reviews and Meta-Analyses extension for Scoping Reviews (PRISMA-ScR), which provides standardized guidance for transparent and reproducible reporting of scoping reviews [8]. The methodological framework was designed to identify and synthesize published evidence describing how pandemic-related and climate-related stressors intersect to affect occupational health, worker vulnerability, and workplace resilience. Although the COVID-19 pandemic represents a central contextual focus of the reviewed literature, the search strategy was intentionally designed to capture broader occupational exposure pathways relevant to routine climate-related hazards and future public health disruptions.

A structured literature search was conducted using PubMed, Scopus, Web of Science, and Google Scholar between April 10 and April 15, 2025. Searches were primarily restricted to English-language peer-reviewed articles published from January 2020 to April 2025, reflecting the period during which combined pandemic-related disruptions and climate-related stressors were most widely documented. One pre-2020 policy study and one SSRN-based source were retained because they met the conceptual relevance criteria described below. The search strategy combined terminology related to occupational health, workplace exposure, pandemic or COVID-19 conditions, and climate or environmental stressors, including extreme heat, air pollution, and environmental exposure. Boolean operators and controlled vocabulary were applied when supported by each database.

Google Scholar was included to capture emerging peer-reviewed studies and interdisciplinary research that may not yet appear in traditional indexing systems, given the evolving nature of research at the intersection of climate hazards and pandemic-related occupational conditions. All retrieved citations were imported into Mendeley for reference management and deduplication. Reference-list screening and forward citation searching were performed to ensure comprehensive coverage. The final search was completed on April 15, 2025.

### 2.2. Study selection and screening

The initial search yielded 612 records. After removal of duplicates, 184 unique articles remained for title and abstract screening. Screening was conducted in two stages. In the first stage, titles and abstracts were reviewed to identify studies addressing occupational populations exposed to both pandemic-related and climate-related stressors, including environmental, organizational, or biological factors. Full-text versions of potentially relevant articles were then assessed to confirm eligibility.

The relatively small final sample reflects the narrow and evolving evidence base specifically addressing the intersection of occupational health, climate-related stressors, and infectious-disease conditions within the same analytical framework. Although many studies independently examined occupational heat stress, environmental exposure, or pandemic-related impacts, substantially fewer studies explicitly or implicitly evaluated how these stressors interact to influence worker health, workplace vulnerability, or occupational resilience.

Although screening and data extraction were conducted primarily by the lead author, supervisory consultation was incorporated as a calibration and quality-control mechanism throughout the review process. Borderline inclusion decisions, study-scope interpretation, and eligibility uncertainties were discussed with the academic supervisor to support consistent application of the predefined inclusion and exclusion criteria. This process helped reduce subjective interpretation during title/abstract screening and full-text eligibility assessment, particularly for studies involving indirect or interdisciplinary occupational exposure pathways.

Ten studies met the inclusion criteria and were retained for synthesis. Common reasons for exclusion included absence of occupational focus, lack of integration between pandemic-related and climate-related exposures, insufficient methodological detail, or publication formats without extractable evidence. The selection process is summarized in the PRISMA-ScR flow diagram (Fig 1). The excluded full-text articles are summarized in S1 Table to improve methodological transparency.

**Fig 1:**
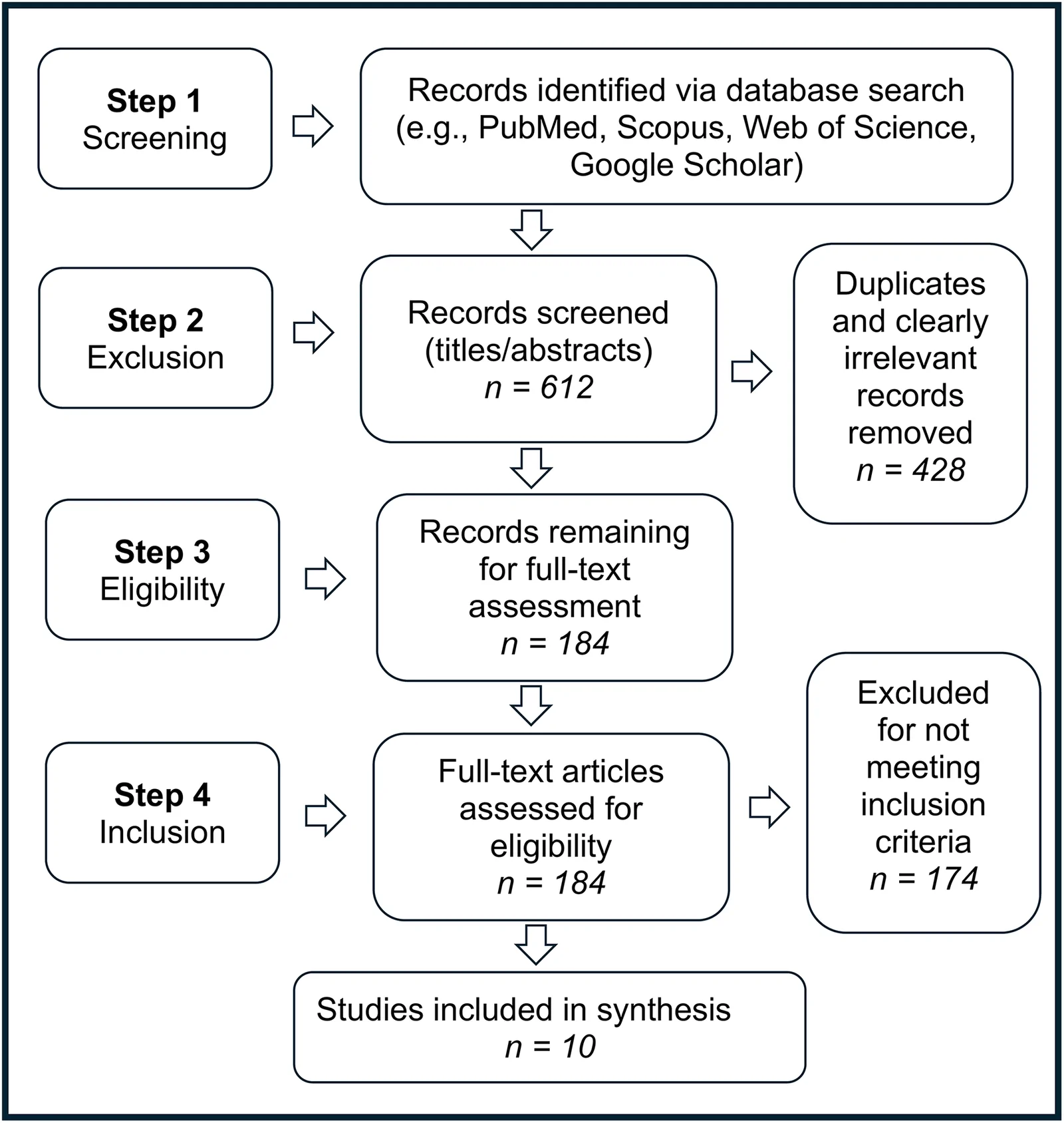
PRISMA-ScR flow diagram of the study selection process. Flow diagram showing the database search, screening, eligibility assessment, and inclusion stages for this scoping review. The complete figure is submitted as a separate figure file in accordance with PLOS ONE figure-submission guidelines.

### 2.3. Eligibility criteria

Studies were eligible for inclusion if they examined occupational populations and addressed both a pandemic-related stressor (e.g., COVID-19 exposure, infectious-disease risk, pandemic-related workplace disruption) and a climate- or environment-related stressor (e.g., extreme heat, wildfire smoke, air pollution, or weather-related exposure). Inclusion criteria emphasized studies that explicitly or implicitly examined interacting exposure pathways, rather than isolated hazards.

Eligible studies were required to present empirical findings, analytical results, or structured syntheses relevant to occupational health, worker vulnerability, or workplace resilience. The review primarily emphasized peer-reviewed and formally structured English-language evidence sources to improve methodological consistency, transparency, and reproducibility. Studies were excluded if they focused solely on non-occupational populations, examined only pandemic-related or only climate-related hazards without meaningful integration of the two domains, lacked sufficient methodological detail, or were commentary-type publications without extractable evidence.

Inclusion criteria prioritized studies examining combined or overlapping exposure pathways related to both climate-related and infectious-disease occupational risks. Studies focusing exclusively on either climate-related hazards or infectious-disease conditions without meaningful integration of the two domains were excluded to maintain conceptual consistency with the objectives of the scoping review.

Although most grey literature sources were excluded, this approach may have limited inclusion of emerging operational reports, institutional guidance documents, and non-indexed occupational-health assessments, particularly in rapidly evolving or resource-limited settings. This limitation is acknowledged in the Discussion.

Two review articles were retained because they synthesized evidence directly relevant to combined occupational risks and contributed meaningfully to the objectives of this scoping review. One pre-2020 policy study was retained because it provided foundational context regarding integration of public health and climate-policy planning frameworks relevant to occupational resilience, preparedness, and multi-sector adaptation. Although this study was published before the COVID-19 pandemic, it was used to support conceptual and governance-related interpretation rather than to represent pandemic-era empirical evidence.

One SSRN-based source was retained because it addressed occupational-health impacts associated with climate-related emergency events and healthcare-system stressors that aligned closely with the objectives of this scoping review. Because SSRN may include preprint or early-access dissemination formats, this source was interpreted cautiously and was not treated as equivalent to high-certainty empirical evidence.

### 2.4. Data charting and extraction

Data extraction followed a structured charting approach using an Excel-based form developed for this review. Extracted variables included study design, geographic location, occupational sector, pandemic-related and climate-related exposures, worker population characteristics, methodological approaches, principal findings, identified vulnerabilities, and reported interventions or policy implications. This approach facilitated comparison across diverse study designs and occupational contexts. All data extraction was conducted by the primary researcher and reviewed with the academic supervisor to ensure accuracy and consistency.

### 2.5. Synthesis approach

Given the heterogeneity of study designs, exposure types, and outcome measures, a thematic synthesis approach was adopted. The synthesis focused on identifying recurring patterns related to compound hazards, vulnerable worker populations, methodological approaches, and gaps in protection or policy. This approach aligns with PRISMA-ScR guidance for mapping the breadth and characteristics of available evidence rather than assessing intervention effectiveness. Because this review followed a scoping-review methodology, no formal risk-of-bias assessment or critical appraisal tool was applied. The purpose of the synthesis was to map the breadth, characteristics, and recurring patterns of the available evidence rather than to determine the comparative strength of evidence, assess intervention effectiveness, or establish causal relationships. A brief methodological quality commentary was incorporated by describing the study designs, data sources, analytical approaches, and key limitations of the included studies. This commentary was intended to help readers interpret the scope and limitations of the evidence base while remaining consistent with PRISMA-ScR objectives.

### 2.6. Protocol and registration

No protocol was registered for this scoping review. Although protocol registration is recommended for systematic reviews, it is not required for scoping reviews. All methodological steps were conducted in accordance with PRISMA-ScR principles to ensure transparency and reproducibility.

### 2.7. Ethics statement

This study is a scoping review based exclusively on previously published and publicly available literature. It did not involve human participants, animal subjects, or identifiable personal data. Therefore, ethical approval was not required.

## 3. Results

Ten included studies or sources met the inclusion criteria, representing a wide spectrum of occupational settings including healthcare, agriculture, construction, emergency response, informal labor, urban pollution-exposed groups, and interdisciplinary occupational-health service contexts. Collectively, these studies demonstrate that climate-related and pandemic-related stressors frequently intersect, amplifying physical, environmental, and psychosocial burdens across diverse work environments. The included studies also extend beyond traditional occupational categories to encompass climate-induced migration, public-health policy integration, and machine-learning–based environmental assessments.

Tables 1–4 present the foundational evidence across these studies. Table 1 summarizes study characteristics, highlighting variation in sector, design, and geographic scope. Table 2 organizes the occupational domains and key thematic areas. Table 3 outlines methodological and analytical approaches, reflecting substantial heterogeneity in study design and technique. Table 4 summarizes vulnerable worker populations and associated risk factors. Together, these tables form a structured map of the evidence landscape and provide the basis for the synthesis presented below.

**Table 1:** Included studies and key characteristics. Summary of study design, sector, and focus for the ten articles analyzed in this review.

Study ID	Full Citation	Sector / Main Focus
**1**	Idris, M. A., Markham, C., Mena, K. D., & Perkison, W. B. (2024). *Examining management and employees’ perceptions of occupational heat exposure and the effectiveness of a heat stress prevention intervention on safety and well-being among natural gas construction workers: A qualitative field-based study. International Journal of Environmental Research and Public Health, 21*(9), 1255. https://doi.org/10.3390/ijerph21091255	Construction – heat stress and prevention interventions
**2**	López-Carr, D., Vanos, J., & Sánchez-Vargas, A. (2022). *Extreme heat and COVID-19: A dual burden for farmworkers. Frontiers in Public Health, 10,* 884152. https://doi.org/10.3389/fpubh.2022.884152	Agriculture – heat exposure and pandemic overlap
**3**	Mehboodi, F., Zamanzadeh, V., & Rahmani, A. (2024). *Occupational safety and health of nurses during the COVID-19 pandemic: The missing part of quality care—A qualitative study. BMJ Open, 14*(6), e083863. https://doi.org/10.1136/bmjopen-2024-083863	Healthcare – nurses’ safety during pandemic
**4**	Tesfaye, A. H., Prior, J., & McIntyre, E. (2025). *Impact of climate change on health workers: A scoping review. Journal of Public Health.* Advance online publication. https://doi.org/10.1007/s10389-025-02418-z	Healthcare – climate impacts on health workforce
**5**	Tsakonas, K., Badyal, S., Takaro, T., & Buse, C. (2025). *Occupational health impacts of climate-related emergency events on health system workers in British Columbia. SSRN Electronic Journal.* https://doi.org/10.2139/ssrn.4954528	Emergency response – health system stress during climate events
**6**	Temirbekov, N., Temirbekova, M., Tamabay, D., Kasenov, S., Askarov, S., & Tukenova, Z. (2023). *Assessment of the negative impact of urban air pollution on population health using machine learning method. International Journal of Environmental Research and Public Health, 20*(18), 6770. https://doi.org/10.3390/ijerph20186770	Urban environment – air pollution and machine learning assessment
**7**	Chirico, F., & Nowrouzi-Kia, B. (2022). *Post-COVID-19 syndrome and new challenges posed by climate change require an interdisciplinary approach: The role of occupational health services. Journal of Health and Social Sciences, 7*(2), 132–136. https://doi.org/10.19204/2022/PSTC1	Occupational health services – post-COVID and climate-related adaptation
**8**	Gupta, D., Kumar, P., Okano, N., & Sharma, A. (2025). *Climate-induced migration in India and Bangladesh: A systematic review of drivers, impacts, and adaptation mechanisms. Climate, 13*(4), 81. https://doi.org/10.3390/cli13040081	Migration – climate-driven displacement and adaptation
**9**	Fox, M., Zuidema, C., Bauman, B., Burke, T., & Sheehan, M. (2019). *Integrating public health into climate change policy and planning: State of practice update. International Journal of Environmental Research and Public Health, 16*(18), 3232. https://doi.org/10.3390/ijerph16183232	Policy – integrating public health and climate planning
**10**	Fan, J., Senthanar, S., Macpherson, R. A., & Sharifian, S. (2021). *An umbrella review of the work and health impacts of working in an epidemic/pandemic environment. International Journal of Environmental Research and Public Health, 18*(13), 6828. https://doi.org/10.3390/ijerph18136828	Cross-sectoral – occupational impacts during pandemics

**Table 2:** Summary of the included studies: occupational domain, focus area, and key themes. Overview of occupational domain, focus area, country context, and recurring themes related to pandemic and climate risks.

Study	Occupational Domain Area	Focus Area	Country/Context	Key Themes
Study 1: Idris et al. (2024)	Heat Stress and Environmental Exposure	Heat stress in construction workers	USA (Texas)	Occupational heat exposure, safety practices, employee perception
Study 2: López-Carr et al. (2022)	Heat Stress and Environmental Exposure	Heat and COVID-19 risks for farmworkers	USA (California) / Mexico	Extreme heat, COVID-19, migrant labor, dual burden
Study 3: Mehboodi et al. (2024)	Heat Stress and Environmental Exposure	Nurse safety during COVID-19	Iran	Personal Protective Equipment (PPE) shortages, occupational safety, qualitative findings
Study 4: Tesfaye et al. (2025)	Heat Stress and Environmental Exposure	Climate change impacts on health workers	Global (> 50 countries)	Physical/mental health, workplace resilience, policy gaps
Study 5: Tsakonas et al. (2025)	Heat Stress and Environmental Exposure	Emergency event risks in healthcare	Canada (British Columbia)	Wildfires, floods, preparedness, health worker safety
Study 6: Temirbekov et al. (2023)	Respiratory Health, Pollution, and Psychological Burden	Machine learning analysis of air pollution and respiratory illness	Kazakhstan	Urban air pollution, respiratory health, machine learning methods
Study 7: Chirico & Nowrouzi-Kia (2022)	Respiratory Health, Pollution, and Psychological Burden	Post-COVID and climate-related occupational health	Global (Italy & Canada)	Post-COVID syndrome, climate adaptation, interdisciplinary solutions
Study 8: Gupta et al. (2025)	Migration, Displacement, and Informal Labor	Climate-induced migration and labor risks	India & Bangladesh	Displacement, informal labor, policy adaptation
Study 9: Fox et al. (2019)	Integrative or Modeling-Oriented Studies	Climate-health policy integration	USA (national framework, policy integration study)	Public health planning, institutional frameworks
Study 10: Fan et al. (2021)	Integrative or Modeling-Oriented Studies	Occupational impacts of epidemics/pandemics	Global (Umbrella Review, > 50 countries)	Pandemic work conditions, occupational health, mitigation strategies

**Table 3:** Methodological and analytical characteristics of the included studies. Study design, data sources, analytical approaches, and limitations illustrating methodological diversity.

Study	Study Design	Data Source	Analytical Method	Key Limitations
Study 1: Idris et al. (2024)	Qualitative field study	Construction worker interviews; field observations	Thematic analysis	Small sample size; limited to one sector
Study 2: López-Carr et al. (2022)	Case study	Farmworker surveys; temperature records	Descriptive comparative analysis	Regional focus; lacks generalizability
Study 3: Mehboodi et al. (2024)	Qualitative interview study	Nurse interviews; COVID-19 experiences	Narrative content analysis	Limited demographic diversity; non-quantitative
Study 4: Tesfaye et al. (2025)	Scoping review	Published literature on climate impacts	Framework-based thematic synthesis	Does not assess real-time field data
Study 5: Tsakonas et al. (2025)	Mixed-methods case analysis	Emergency service data; climate event records	Integrated risk mapping	Lacks long-term outcome evaluation
Study 6: Temirbekov et al. (2023)	Modeling-based empirical study	Air quality data; respiratory illness records	Machine learning algorithms; correlation models	Model performance not validated externally
Study 7: Chirico & Nowrouzi-Kia (2022)	Editorial with interdisciplinary synthesis	Published reports and expert opinion	Conceptual framing; qualitative synthesis	Not empirical; no new data
Study 8: Gupta et al. (2025)	Systematic review	Scopus-indexed papers on migration	Systematic literature mapping	Geographically limited; publication bias
Study 9: Fox et al. (2019)	Policy review	Public health planning documents	Policy evaluation using CFES model	Conceptual model only; not implementation-based
Study 10: Fan et al. (2021)	Umbrella review	Review articles on occupational pandemics	Narrative synthesis of review articles	No meta-analysis; limited intervention focus

**Table 4:** Occupational vulnerabilities highlighted in the included studies. Worker populations identified as most vulnerable, their risk factors, and proposed interventions.

Study	Vulnerable Population	Risk Factors Identified	Suggested Policy or Intervention
Study 1: Idris et al. (2024)	Construction workers	Heat exposure, dehydration, PPE discomfort	Hydration stations, adjusted work shifts, PPE modifications
Study 2: López-Carr et al. (2022)	Migrant farmworkers	Extreme heat, dual pandemic risk, limited protection	Cooling shelters, targeted aid, inclusive OSH plans
Study 3: Mehboodi et al. (2024)	Nurses in clinical settings	PPE shortages, mental stress, lack of policy inclusion	Mental health programs, PPE access, training
Study 4: Tesfaye et al. (2025)	Global health workers	Workforce burnout, systemic gaps, resource limits	Global workforce protection frameworks
Study 5: Tsakonas et al. (2025)	Emergency health responders	Wildfire exposure, disaster response fatigue	Climate emergency preparedness training and support
Study 6: Temirbekov et al. (2023)	Urban populations in polluted cities	Chronic air pollution, respiratory and mental health risks	Urban emission controls, health tracking tech
Study 7: Chirico & Nowrouzi-Kia (2022)	PostCOVID-19 survivors and workers	Psychological fatigue, lack of interdisciplinary care	Multidisciplinary OSH policy integration
Study 8: Gupta et al. (2025)	Displaced and informal laborers	Forced migration, housing loss, income insecurity	Legal protection, relocation support, livelihood aid
Study 9: Fox et al. (2019)	Public health officials and planners	Fragmented systems, lack of occupational inclusion	Interagency integration, resilience-based governance
Study 10: Fan et al. (2021)	Essential workers across sectors	Occupational exposure during public health emergencies	Cross-sector OSH training, resource distribution

Tables 1–4 are embedded within the main manuscript as core evidence-synthesis tables. They are not presented as supplementary materials because they summarize the study characteristics, occupational domains, methodological approaches, and vulnerable worker populations that form the basis of the scoping review findings.

### 3.1. Study characteristics

Table 1 illustrates substantial diversity across the ten included studies, spanning construction, agriculture, nursing and healthcare, emergency response, urban pollution environments, interdisciplinary occupational-health services, climate-induced migration, and public-health policy integration. The studies originate from the United States, Canada, Iran, Kazakhstan, India, Bangladesh, and multi-country global contexts, reflecting a broad geographic range.

Research designs vary considerably, including qualitative field-based interviews, case studies, systematic and scoping reviews, mixed-methods case analyses, machine-learning environmental modeling, policy evaluations, interdisciplinary conceptual analyses, and umbrella reviews synthesizing pandemic-related occupational impacts. This mix of empirical and conceptual evidence shows that climate- and pandemic-related occupational risks are being examined from multiple disciplinary angles, though with uneven methodological depth. While qualitative studies provide detailed insights into worker experiences, review-based and modeling-oriented studies contribute broader systemic and environmental perspectives.

### 3.2. Occupational domains, focus areas, and key themes

Table 2 organizes the included studies into four major occupational domains, each highlighting distinct but interrelated stressor patterns. The first domain heat stress and environmental exposure represents the largest cluster and includes construction workers, migrant farmworkers, nurses in Personal Protective Equipment (PPE), global health workers, and emergency responders. These studies consistently show that extreme heat, wildfire smoke, and disaster-related environmental conditions intersect with pandemic-related workloads, producing dual burdens that heighten fatigue, dehydration, and injury risk in both outdoor and indoor settings.

The second domain focuses on respiratory health, air pollution, and psychological burdens, including machine-learning analyses of urban air-quality exposures and conceptual assessments of post-COVID occupational-health service needs. These studies reveal that chronic pollution exposure, wildfire smoke, and lingering post-infection symptoms disproportionately affect workers with high baseline occupational strain, underscoring the need for integrated, interdisciplinary support.

The third domain covers migration, displacement, and informal labor, emphasizing the compounded vulnerabilities faced by climate-induced migrants and precarious workers in India and Bangladesh. These populations experience unstable housing, limited legal protections, high outdoor exposure, and constrained access to protective infrastructure.

The fourth domain includes integrative and policy-oriented studies, such as national climate-health planning assessments and umbrella reviews of pandemic-related occupational impacts. These studies point to systemic issues including fragmented governance, inadequate cross-sector coordination, and limited incorporation of occupational health into climate and public-health frameworks. Together, the four domains illustrate how climatic, biological, and socioeconomic pressures converge across occupations, often reinforcing inequities and revealing broad gaps in preparedness.

### 3.3. Methodological and analytical approaches

Table 3 highlights substantial methodological heterogeneity across the included studies. The dataset includes qualitative interviews, case studies, systematic reviews, scoping reviews, mixed-methods analyses, machine-learning–based modeling, policy evaluations, and interdisciplinary conceptual syntheses. These differing designs reflect the emerging and multidimensional nature of research on combined climate- and pandemic-related occupational risks.

Data sources range from worker interviews and field observations to farmworker surveys, temperature and climate-event records, respiratory illness databases, air-pollution datasets, migration literature, and public-health policy documents. Review-based studies draw from large international evidence pools, while empirical studies rely on localized or sector-specific data.

Analytical methods vary accordingly. Qualitative studies use thematic or narrative content analysis, while mixed-methods studies incorporate integrated risk mapping. The machine-learning study applies predictive modeling to link air pollution with respiratory outcomes, and policy evaluations employ frameworks such as the Climate-Focused Environmental Strategies (CFES) model. Systematic and umbrella reviews utilize structured mapping and narrative synthesis. Despite this breadth, common limitations include small sample sizes, regional focus, limited demographic diversity, lack of longitudinal data, absence of external validation in modeling studies, and conceptual (rather than implementation-based) policy analyses. These gaps underscore the need for more harmonized, integrated, and data-rich research approaches.

### 3.4. Occupational vulnerabilities and risk factors

Table 4 identifies a wide range of vulnerable worker populations, including construction workers, migrant farmworkers, nurses, global health workers, emergency responders, urban pollution-exposed populations, post-COVID-19 survivors, displaced and informal laborers, public-health officials and planners, and essential workers across sectors. These groups experience heightened exposure due to both workplace conditions and broader socioeconomic factors.

Recurring risk factors include extreme heat, dehydration, PPE-related discomfort, dual exposure to infectious disease and environmental hazards, burnout, wildfire smoke, disaster-related fatigue, chronic air pollution, forced migration, housing instability, and limited access to institutional protections. Structural vulnerabilities such as fragmented governance, insufficient occupational-health infrastructure, and inadequate policy integration further amplify these risks.

Proposed interventions include hydration stations, cooling shelters, adjusted work shifts, PPE redesign, enhanced mental-health support, global workforce protection frameworks, climate-emergency preparedness training, urban emission controls, health-tracking technologies, interdisciplinary coordination, legal protections for migrant and informal workers, and resilience-based governance models. Taken together, these recommendations highlight the need for both immediate workplace-level strategies and broader systemic reforms.

### 3.5. Summary of evidence and cross-table synthesis

Synthesis of findings across Tables 1–4 reveals several overarching and interrelated patterns in how climate-related stressors and infectious-disease conditions shape occupational health risks across sectors and geographic contexts.

The findings summarized in this section represent evidence-derived patterns identified across the included studies. These patterns include recurring occupational vulnerabilities, overlapping exposure pathways, methodological limitations, and policy fragmentation. Because the included evidence is heterogeneous and largely descriptive, these findings should be interpreted as thematic synthesis rather than as formal evidence grading or causal inference. Broader interpretation, policy implications, and future directions are addressed separately in the Discussion to reduce overlap between Results and Discussion.

First, the evidence consistently demonstrates that multidimensional hazards, including extreme heat, wildfire smoke, infectious-disease exposure, and psychosocial strain, frequently co-occur within occupational settings. These interacting stressors operate through shared exposure pathways, producing cumulative risks that exceed those associated with individual hazards alone. Such patterns are observed across diverse occupations, including agriculture, construction, healthcare, emergency response, and informal labor environments.

Second, the synthesis highlights the central role of social and structural inequities in shaping occupational vulnerability. As summarized in Tables 2 and 4, migrant workers, informal laborers, seasonal and cross-border workers, older adults, women, and low-income populations are consistently exposed to disproportionate risks. These inequities are reinforced by limited access to healthcare, constrained legal and regulatory protections, inadequate cooling and ventilation infrastructure, and reduced capacity to modify work schedules or conditions. Across the included studies, vulnerability emerges not solely from environmental exposure but from the interaction of employment precarity, housing instability, and governance gaps.

Third, cross-table comparison reveals substantial methodological fragmentation within the current evidence base. As outlined in Table 3, most studies rely on qualitative, descriptive, or cross-sectional designs that characterize existing occupational risks but provide limited capacity to assess cumulative exposure, temporal dynamics, or future scenarios. Advanced analytical approaches, including predictive modeling, geospatial analysis, and integration of climate, occupational, and health datasets, remain underutilized and unevenly distributed across regions and sectors. This heterogeneity constrains comparability across studies and limits the development of anticipatory occupational-health strategies.

Finally, the synthesis underscores persistent gaps in policy integration and implementation. Although many studies identify practical interventions, such as hydration access, cooling strategies, ventilation improvements, personal protective equipment modification, and mental-health support, these measures are frequently addressed in isolation rather than within coordinated, multi-hazard frameworks. Fragmented governance structures and sector-specific policy silos hinder the translation of evidence into comprehensive occupational protections. Collectively, the evidence synthesized across Tables 1–4 underscores the need for integrated, equity-focused, and forward-looking occupational-health approaches that align climate adaptation, infection prevention, and workforce resilience planning.

## 4. Discussion

This scoping review examined how climate-related stressors and infectious-disease conditions intersect to influence occupational health across diverse sectors and geographic contexts. Although the COVID-19 pandemic provided a unique and well-documented global disruption, the evidence synthesized here demonstrates that the mechanisms underlying compounded occupational risk such as heat exposure, poor air quality, workload intensification, and psychosocial strain, extend well beyond pandemic-specific circumstances. Rather than representing a temporary or isolated phenomenon, the pandemic functioned as a large-scale stress test that exposed structural vulnerabilities already present in many work environments.

To maintain appropriate alignment between the available evidence and the interpretation of findings, the Discussion distinguishes between evidence-derived findings and forward-looking policy implications. Findings directly supported by the reviewed studies are presented as recurring themes, including compound exposure pathways, vulnerable worker populations, methodological gaps, and fragmented occupational-health policies. Broader recommendations related to predictive analytics, interoperable data platforms, early-warning systems, and integrated governance are presented as future-oriented considerations informed by identified gaps rather than as established or empirically validated interventions.

Across the ten studies included in this review, climate and infectious hazards consistently interacted to amplify physical, environmental, and psychosocial pressures on workers. As summarized in Table 2, these interactions occurred through overlapping pathways including extreme heat, wildfire smoke, inadequate ventilation, PPE-related thermal burden, and intensified job demands. Importantly, many of these exposure pathways are routinely encountered in non-pandemic contexts, particularly in healthcare, emergency response, construction, agriculture, and other high-exposure occupations where personal protective equipment, long shifts, and environmental stressors are part of standard operations.

The synthesis presented in Tables 2–4 indicates that the burden of these compounded risks is not evenly distributed. Workers in low-income, migrant, informal, and seasonal employment arrangements experience heightened exposure due to limited regulatory protections, constrained access to cooling infrastructure, and reduced capacity to modify work schedules or environments. These inequities persist regardless of pandemic status and reflect broader structural determinants of occupational vulnerability, reinforcing the relevance of integrated, multi-hazard frameworks for worker protection.

From a methodological perspective, the findings summarized in Table 3 highlight persistent limitations in the way combined climate and health risks are currently studied. Most investigations rely on descriptive or cross-sectional designs that characterize existing exposures but do not adequately model cumulative or future risk. This constrains the ability to anticipate compound hazards during extreme heat events, wildfires, air-pollution episodes, or future infectious-disease outbreaks, all of which are increasingly recognized within occupational health and industrial hygiene practice.

Taken together, the findings support the conceptual framing of this scoping review by showing that occupational-health risks emerge from overlapping environmental, biological, social, and policy-related determinants rather than from isolated hazards alone. Because this review was designed to map evidence rather than test hypotheses, these patterns should be interpreted as analytical themes identified across the reviewed literature rather than as formally tested causal claims. While pandemic conditions intensified many of the risks observed, the pathways identified are broadly applicable to routine heat stress management, disaster preparedness, and workforce resilience planning. In this context, existing occupational heat-stress guidelines and regulatory frameworks provide an essential foundation for mitigation, yet the reviewed literature suggests that their application remains uneven across sectors and regions. The following subsections (4.1–4.7) expand on six key themes emerging from the synthesis: (1) compound hazards, (2) inequitable exposure patterns, (3) research and data gaps, (4) policy fragmentation, (5) integrated mitigation strategies, and (6) broader implications for workforce resilience.

### 4.1. Compound hazards and health effects

Many occupational settings are routinely exposed to overlapping environmental and work-related stressors that intensify injury and illness risks, particularly when climatic extremes coincide with periods of heightened operational demand or public health disruption. As summarized in Table 2, outdoor laborers in agriculture, construction, and transportation frequently experience extreme heat, poor air quality, and elevated physical workload while also adhering to infection-prevention measures during outbreak scenarios. These combined pressures increase thermal strain, dehydration, cardiovascular load, and fatigue, especially among workers performing high-intensity tasks in direct sunlight or while wearing protective equipment that restricts heat dissipation [11,12]. Evidence from construction and industrial sectors demonstrates that comparable physiological stress patterns occur even outside pandemic contexts, underscoring the broader relevance of structured heat-stress prevention programs and engineering controls [13].

In tropical and subtropical regions, recurring heat waves already constrain safe work hours and limit physiological recovery time. During periods of large-scale disruption such as pandemics, extreme weather events, or emergency response operations, these constraints are further intensified by reduced access to shaded rest areas, hydration points, and adequately ventilated workspaces [14,15]. As shown in Table 2, sector-specific reviews consistently report that adaptation measures including scheduled breaks, cooling infrastructure, ventilation improvements, and redesigned PPE remain unevenly implemented across regions and industries, regardless of whether exposures occur under routine or crisis conditions [1,11].

The interaction of physical and psychosocial stressors further compounds occupational health risks. Long shifts, high task demands, staffing shortages, and prolonged exposure to hazardous environments contribute to elevated cardiovascular strain, impaired cognitive functioning, and increased accident risk [12,15]. Multi-sector syntheses indicate that coordinated interventions such as rest-break scheduling, ventilation optimization, task rotation, and targeted educational programs can substantially mitigate heat stress while preserving productivity and safety across a range of occupational contexts [1,13].

These compound pressures disproportionately affect low-income, migrant, and informal outdoor workers highlighted in Table 4, who often lack stable employment protections, access to cooling infrastructure, or institutional oversight [14,15]. In such settings, cumulative exposures are shaped not only by environmental conditions but also by social and organizational determinants, including job insecurity, limited access to healthcare, and constrained recovery opportunities. Future research and practice would benefit from integrating wearable sensors, real-time physiological monitoring, and predictive heat-strain modeling to better characterize cumulative exposure pathways and support locally tailored prevention strategies across both routine operations and crisis conditions [11].

### 4.2. Inequitable risk among vulnerable workers

Occupational risks arising from overlapping climate-related and infectious-disease stressors are not evenly distributed across worker populations. As shown in Table 4, migrant laborers, informal and part-time employees, and seasonal or cross-border agricultural workers are consistently exposed to physically demanding tasks under extreme heat, degraded air quality, and limited access to rest areas or cooling infrastructure. These conditions increase chronic dehydration, thermal strain, and infection risk, particularly in settings where safety standards are inconsistently applied or fail to account for the needs of diverse worker groups [16]. Meta-analyses further demonstrate that heat-related injuries rise sharply among agricultural and construction workers in low-income regions where long work hours intersect with restricted access to shade, water, or temperature-controlled environments [17,18].

Workers engaged in temporary, informal, or precarious employment arrangements often lack health insurance, paid leave, or union representation, limiting their ability to report unsafe conditions or recover from illness without economic penalty. Displaced and homeless workers highlighted in Table 4 experience additional vulnerabilities related to unstable housing, inadequate climate control, and restricted access to sanitation and clean water [19]. Older adults face heightened susceptibility to extreme heat due to age-related changes in thermoregulation and higher prevalence of chronic disease, increasing risks of cognitive impairment, injury, and heat-related mortality during periods of sustained exposure [20]. Women across multiple sectors experience compounded burdens associated with occupational exposure and unpaid caregiving responsibilities, which intensify stress and reduce opportunities for rest and recovery [16].

Evidence summarized in Table 4 indicates that targeted interventions can meaningfully reduce these inequities when appropriately implemented. Heat-acclimatization programs, micro-rest breaks, hydration stations, equitable scheduling practices, and culturally and linguistically accessible communication materials are associated with lower injury rates and improved safety compliance among vulnerable worker populations [17,21]. Studies involving displaced and low-income groups further demonstrate that low-cost measures such as community cooling centers, emergency vouchers, and accessible hydration access points improve resilience during concurrent climate-related and public health stressors [19].

Personalized heat-risk communication strategies, wearable monitoring technologies, and community-based alert systems have also been shown to reduce heat-related emergency visits among older adults and high-risk workers, including during periods of heightened operational strain [20]. Together, these findings highlight that occupational vulnerability is shaped not only by environmental exposure but also by legal status, economic security, access to protective infrastructure, and inclusion within policy frameworks. Addressing inequitable risk therefore requires integrated approaches that combine workplace-level protections with broader social and institutional support mechanisms.

### 4.3. Gaps in research and data

As summarized in Table 3, current research on combined climate-related and infectious-disease occupational risks remains constrained by methodological and data limitations that restrict generalizability and future preparedness. Most studies rely on descriptive or cross-sectional designs that characterize exposures and health outcomes at a single point in time but lack the capacity to model cumulative risk, assess long-term impacts, or anticipate future hazard scenarios. This continued reliance on static approaches limits understanding of how interacting stressors such as extreme heat, air pollution, and infectious-disease exposure evolve across seasons, regions, and occupational settings [22].

Emerging advances in geospatial analysis, remote sensing, and environmental modeling show considerable promise for improving occupational risk assessment, yet these tools remain underutilized in the studies reviewed. Only a small number of investigations integrate satellite-derived climate data, ground-based environmental monitoring, or spatial overlays of worker locations, despite strong evidence that spatially explicit approaches can more accurately characterize heat exposure, identify microclimate variability, and inform targeted workplace interventions [23]. This gap is particularly evident in agriculture, construction, and outdoor service sectors highlighted in Table 2, where environmental conditions can vary substantially within short distances and timeframes.

Data fragmentation further constrains analytical progress. Health records, meteorological data, occupational injury reports, and workforce characteristics are often collected and managed in separate systems, limiting interoperability and real-time risk tracking. Although machine-learning based approaches have demonstrated potential for linking environmental indicators to health outcomes, these methods remain concentrated in high-income settings and are rarely applied to informal or resource-limited labor contexts [24]. As shown in Table 3, many modeling studies also lack external validation or longitudinal follow-up, reducing confidence in their predictive utility.

Cross-national comparability remains limited by inconsistent exposure metrics, reporting standards, and regulatory thresholds. Although international research increasingly calls for harmonized approaches to occupational heat-stress assessment, empirical implementation varies widely across countries and sectors, hindering meaningful comparison and synthesis [25]. These inconsistencies are especially consequential for migrant and transboundary worker populations identified in Table 4, whose exposures often span multiple regulatory environments.

Addressing these gaps will require coordinated investment in interdisciplinary research, interoperable data infrastructures, and predictive analytics that integrate environmental, physiological, and organizational data. Strengthening collaboration among climate scientists, engineers, occupational-health practitioners, and policymakers can support the development of early-warning systems and adaptive risk-management tools applicable across diverse settings [26]. These limitations in research design and data integration underscore the need for more cohesive policy frameworks and management strategies, which are examined in the following section.

### 4.4. Policy integration challenges and management limitations

Occupational safety frameworks continue to address climate-related hazards and infectious-disease threats through largely separate policy channels, resulting in fragmented guidance that inadequately protects workers during overlapping crises. As summarized in Table 2, climate-adaptation measures typically emphasize hydration, shade, rest breaks, and ventilation, while infectious-disease protocols prioritize distancing, isolation, and personal protective equipment. When implemented independently, these measures may conflict at the operational level. For example, protective equipment designed for infection control can exacerbate heat stress among outdoor and high-exertion workers, while distancing requirements may limit access to shaded rest areas or cooling spaces [27]. These challenges underscore the limitations of siloed policy approaches when environmental and biological hazards co-occur.

Established occupational-health authorities have long recognized heat stress as a core workplace hazard and provide guidance addressing exposure thresholds, work–rest cycles, hydration, acclimatization, and environmental controls. Guidance issued by Occupational Safety and Health Administration (OSHA), the National Institute for Occupational Safety and Health (NIOSH), and the World Health Organization (WHO) emphasizes prevention strategies that are broadly applicable across sectors and geographic regions. However, the evidence synthesized in this review indicates that such guidance is unevenly implemented, inconsistently enforced, or insufficiently adapted to account for concurrent infectious-disease risks, particularly in informal, seasonal, and resource-limited work settings.

Legal and regulatory frameworks also play an important role in protecting workers from combined climate-related and infectious-disease occupational risks. National and international occupational safety systems emphasize employer responsibilities to identify hazards, assess workplace risks, implement preventive controls, and provide safe and healthy working conditions. Integrating climate adaptation, infection prevention, heat-stress management, ventilation, PPE planning, and psychosocial support into occupational risk assessment can strengthen compliance and improve worker protection across routine operations and emergency conditions.

Sectoral evidence from manufacturing, construction, healthcare, energy, and emergency response demonstrates the value of coordinated adaptation strategies when climate and public-health contingencies are addressed together. Some organizations have adopted integrated emergency-management systems that align ventilation standards, PPE specifications, scheduling practices, and hydration requirements under unified occupational-risk frameworks [28]. Occupational-health professionals increasingly advocate for harmonized policies that minimize contradictory guidance and reduce compliance burdens while maintaining worker safety across diverse exposure scenarios [29]. Nevertheless, as highlighted in Table 4, small enterprises, informal employers, and migrant labor contexts often fall outside formal regulatory oversight and lack the institutional capacity or financial resources required for comprehensive implementation.

Data and governance fragmentation further compound these challenges. Weak integration across health surveillance, meteorological monitoring, labor inspection, and environmental reporting systems highlighted in Table 3 limits the ability to track compound risks in real time or to coordinate timely interventions. Inconsistent heat-stress thresholds, variable reporting standards, and jurisdiction-specific regulatory mandates impede translation of scientific evidence into coherent workplace policy [25,30]. Comparable concerns have been raised in European occupational-health contexts, where guidance from bodies such as the European Agency for Safety and Health at Work (EU-OSHA) highlights the need for cross-sector coordination but faces similar implementation barriers across member states.

Addressing these policy integration gaps requires governance models that explicitly recognize compound hazards and promote coordination across occupational health, public health, environmental protection, and emergency management. Cross-sector approaches informed by One Health principles can support early warning, streamline risk communication, and align preventive measures across environmental, occupational, and epidemiological domains [31]. Modernizing occupational-safety systems to incorporate predictive analytics, interoperable data platforms, and performance-based indicators as recommended by Ebi et al. (2021) and Neta et al. (2022) would further strengthen preparedness and equity. Without such integration, existing policies risk remaining reactive and fragmented, limiting their effectiveness in protecting workers during future climate-driven and infectious-disease crises. Implementation feasibility varies substantially across regulatory and economic contexts, particularly in informal and low-resource labor settings.

### 4.5. Integrated strategies and future directions

The evidence synthesized in this review highlights the importance of integrated, multi-hazard approaches that address the combined effects of climate stressors, infectious-disease risks, and psychosocial pressures on worker health. As summarized in Table 2, many occupational settings continue to rely on fragmented safety practices that address hazards individually rather than as interacting systems. This separation limits effectiveness during periods of concurrent stress, particularly in sectors characterized by high physical demand, outdoor exposure, or prolonged operational strain.

The strategies discussed in this section should be interpreted as forward-looking implications rather than confirmed measures of intervention effectiveness. The reviewed studies identify recurring needs for integrated surveillance, adaptive risk management, worker-centered communication, and cross-sector coordination; however, most do not directly evaluate these strategies through longitudinal or intervention-based designs. Therefore, recommendations regarding early-warning systems, predictive analytics, interoperable data platforms, and integrated governance should be understood as evidence-informed directions for future occupational-health planning and research.

Across the reviewed studies, several intervention strategies emerge as consistently beneficial across both routine and crisis conditions. These include adaptive work–rest scheduling, access to hydration and cooling infrastructure, ventilation improvements, task rotation, and modifications to protective equipment to reduce thermal burden [1, 32]. Importantly, these measures are not limited to pandemic contexts; they also apply to heat waves, wildfire events, and other climate-driven disruptions that increasingly affect occupational environments. When implemented in combination rather than isolation, such strategies demonstrate greater potential to reduce heat-related illness, fatigue, and injury while maintaining productivity.

Technological innovations offer additional opportunities to support integrated risk management. As shown in Tables 2 and 3, wearable sensors, mobile health platforms, and environmental monitoring tools can provide real-time feedback on physiological strain, ambient temperature, air quality, and workload [33,34]. When paired with clear communication pathways and worker-centered design, these tools support early detection of risk, timely intervention, and continuous adaptation to changing conditions. However, their effectiveness depends on equitable access, data privacy protections, and integration into broader occupational-health systems rather than standalone deployment.

Worker participation is an important component of effective occupational-risk prevention, particularly when climate-related hazards and infectious-disease conditions overlap. Participatory safety planning, worker engagement, union representation where applicable, and participatory management approaches can strengthen risk identification, improve communication, support trust, and increase the feasibility of protective measures. Evidence summarized in Table 4 indicates that involving workers in the design, testing, and refinement of prevention strategies improves uptake, compliance, and trust across diverse occupational settings [1,35]. Worker engagement also helps ensure that interventions are culturally appropriate, operationally feasible, and responsive to real-world constraints, particularly for migrant, informal, seasonal, and low-income workers, whose perspectives may otherwise be underrepresented in workplace safety planning. Integrating physical-risk mitigation with psychosocial support and mental-health resources provides a more holistic foundation for resilience during prolonged or recurrent stressors.

Looking forward, occupational-health systems must evolve from reactive, hazard-specific models toward adaptive frameworks capable of anticipating compound risks. Aligning climate adaptation, public-health preparedness, and digital surveillance efforts can support coordinated responses to emerging threats and reduce reliance on ad hoc crisis management [36,37]. Strengthening interdisciplinary collaboration, investing in workforce training, and improving translation of research into practice will be essential for ensuring that integrated strategies are scalable and equitable across sectors and regions.

### 4.6. Broader implications for workforce resilience

The convergence of climate-related and infectious-disease stressors underscores the need for systems-oriented approaches to workforce resilience that integrate physical, environmental, organizational, and psychosocial dimensions of worker well-being. As summarized in Table 2, many interventions originally developed for infection control such as improved ventilation, workspace redesign, and reduced indoor crowding also provide co-benefits for thermal comfort, air quality, and overall occupational health. Adopting such dual-benefit strategies allows organizations to maximize protective impact while promoting consistency and sustainability across diverse work environments [2,38].

Long-term workforce resilience depends not only on technical controls but also on organizational flexibility and social protection mechanisms. Evidence across multiple sectors indicates that adaptive scheduling, climate-responsive work–rest cycles, and staffing policies that accommodate extreme environmental conditions reduce injury risk, absenteeism, and burnout [39,40]. These measures are particularly important for workers in high-exposure or precarious employment arrangements, including migrant, informal, and low-income populations highlighted in Table 4, who often lack the financial or legal flexibility to recover from compounded stressors.

Psychosocial resilience represents an additional and often under-addressed dimension of occupational health. Studies involving healthcare, emergency response, and public-sector workers emphasize that peer support networks, team-based safety planning, stress-management training, and clear, multilingual communication enhance coping capacity during prolonged or recurrent crises [41]. When combined with physical risk mitigation, these approaches support sustained performance and reduce long-term health consequences associated with chronic occupational strain.

Workforce resilience also requires alignment across institutional and governance levels. Cross-sector coordination among health, labor, environmental, and emergency-management agencies improves preparedness by ensuring consistent guidance, shared situational awareness, and coordinated resource allocation. As shown in Table 3, fragmentation across surveillance systems and policy domains limits the ability to anticipate emerging risks or respond effectively to compound hazards. Strengthening interoperability, data sharing, and collaborative planning can therefore enhance resilience at both organizational and societal scales.

Investing in workforce resilience yields measurable public-health and economic benefits. Reductions in heat-related illness, infectious-disease transmission, turnover, and productivity loss offset the costs of preventive interventions and contribute to organizational continuity during periods of disruption. These outcomes align with global priorities such as SDG 3 (Good Health and Well-Being) and SDG 8 (Decent Work and Economic Growth), reinforcing the importance of protecting workers as a core component of sustainable development [2,42]. Collectively, the evidence indicates that resilient occupational systems are not only better equipped to manage future crises but also support healthier, safer, and more equitable work environments under routine conditions.

### 4.7. Limitations

This review has several limitations. First, the final number of included studies was small, reflecting the emerging nature of research specifically addressing combined climate-related and infectious-disease occupational risks. Second, screening and data extraction were conducted primarily by one reviewer, although supervisory consultation was used to support calibration of borderline decisions and consistency in applying eligibility criteria; independent dual-reviewer screening was not feasible. Third, exclusion of most grey literature may have limited inclusion of emerging operational reports, institutional guidance, or non-indexed occupational-health assessments. Fourth, the included studies were heterogeneous in design, geographic scope, occupational focus, data source, and analytical approach, limiting direct comparison across studies. Finally, the reviewed evidence remains largely descriptive, cross-sectional, conceptual, or review-based, with limited longitudinal or intervention-based research available to evaluate cumulative exposure, causal pathways, or policy effectiveness.

The inclusion of one pre-2020 policy study and one SSRN-based source should also be considered when interpreting the findings. The 2019 policy study was included for foundational context related to climate–public health integration, while the SSRN-based source was included because of its relevance to climate-related emergency events and healthcare-system occupational stressors. However, non-indexed or preprint-based evidence may not have undergone the same level of peer-review scrutiny as fully indexed journal articles. Therefore, these sources were interpreted cautiously and used primarily to support contextual mapping rather than conclusions about intervention effectiveness or causal relationships.

The findings of this scoping review should be interpreted as evidence mapping rather than as a formal assessment of evidence strength. The included studies varied substantially in design, geographic scope, occupational setting, data source, and analytical approach. Many studies were qualitative, descriptive, conceptual, cross-sectional, or review-based, which limits direct comparison across studies and restricts conclusions regarding intervention effectiveness, predictive certainty, or causal relationships. Therefore, policy and resilience-related implications presented in this review should be understood as forward-looking interpretations informed by recurring themes and identified gaps rather than as conclusions supported by formal critical appraisal or intervention-effectiveness assessment.

### 4.8. Conclusions

This scoping review shows that climate-related and infectious-disease stressors commonly occur together in occupational settings, resulting in compounded physical, environmental, and psychosocial risks that extend beyond the scope of traditional single-hazard safety approaches. Across diverse sectors, these interacting stressors were associated with increased fatigue, thermal strain, respiratory burden, and psychological stress, particularly in work environments characterized by high physical demands, prolonged shifts, and exposure to extreme environmental conditions.

The findings also indicate that occupational risks linked to combined climate and infectious hazards are not evenly distributed. Workers in migrant, informal, seasonal, and low-income employment, as well as older workers and women, experienced disproportionate vulnerability due to limited access to healthcare, cooling and ventilation infrastructure, stable employment protections, and institutional support. These patterns reflect broader structural and social determinants of occupational health rather than isolated environmental or biological exposures.

From a methodological perspective, the reviewed literature remains largely descriptive, with limited use of longitudinal designs, predictive modeling, or integrated environmental and occupational datasets. This constrains the ability to assess cumulative exposure, anticipate future risk scenarios, or support proactive occupational health planning in the context of increasing climate variability and recurring public health disruptions.

At the same time, the evidence points to important opportunities for improving occupational resilience. Integrated approaches that jointly address climate adaptation, infection prevention, and psychosocial well-being may provide practical benefits across both routine operations and crisis conditions. Measures such as adaptive work scheduling, improved ventilation, access to hydration and cooling, participatory safety planning, and data-informed monitoring systems may help reduce injury and illness while supporting workforce continuity; however, these strategies require further evaluation through longitudinal, intervention-based, and implementation-focused research.

Overall, the conclusions of this review reflect evidence mapping and thematic synthesis rather than formal evidence grading or causal assessment. The review identifies recurring patterns and gaps across the available literature and uses these findings to propose evidence-informed directions for integrated occupational-health preparedness, workforce resilience, and multi-hazard policy development. The findings should be interpreted with caution because of the limited number of available studies combining climate-related and infectious-disease occupational risks, heterogeneous study designs, and the emerging nature of the evidence base. Nevertheless, the review highlights the need for occupational health systems to move beyond reactive, hazard-specific models toward more anticipatory and systems-oriented frameworks that account for intersecting environmental and public health risks. Strengthening cross-sector coordination, improving data integration, and embedding equity considerations into occupational policy may help protect vulnerable worker populations and enhance preparedness in a changing climate. By jointly examining pandemic-related and climate-related stressors within occupational contexts, this review contributes evidence to support more integrated and forward-looking strategies for safer and more resilient workforces.

## Supporting information

S1 Checklist PRISMA-ScR checklist. This checklist summarizes how the scoping review addresses the Preferred Reporting Items for Systematic Reviews and Meta-Analyses extension for Scoping Reviews (PRISMA-ScR) reporting items.
(DOCX)

S1 Table Full-text articles excluded after eligibility assessment and primary reasons for exclusion. This table summarizes excluded full-text studies and the primary reasons for exclusion, including absence of occupational focus, lack of integrated climate-related and infectious-disease analysis, insufficient methodological detail, or publication types without extractable evidence.
(XLSX)

S1 Fig Conceptual framework of climate-related and infectious-disease occupational-health pathways, worker vulnerabilities, health outcomes, and resilience strategies. The framework summarizes how environmental hazards, biological risks, psychosocial strain, and structural vulnerabilities may interact across occupational settings and highlights opportunities for integrated prevention, preparedness, and policy response.
(PPTX)

## Abbreviations

OSH: Occupational Safety and Health
PPE: Personal Protective Equipment
ML: Machine Learning
PM2.5: Particulate Matter less than 2.5 micrometers
SDG: Sustainable Development Goal
