# Supplementary material for: Occupational health risks from climate-related and infectious-disease stressors: A scoping review of worker safety and resilience
Source: PLoS One. 2026 Oct 21;21(10):e0361296. doi: 10.1371/journal.pone.0361296 (PMC13686495; doi:10.1371/journal.pone.0361296)
Supplement: Supplementary file 1 [file pone.0361296.s001.docx]

**PRISMA-ScR Checklist**

*(Preferred Reporting Items for Systematic Reviews and Meta-Analyses extension for Scoping Reviews)*

**Manuscript Title:** Occupational Health Risks from Climate-Related and Infectious-Disease Stressors: A Scoping Review of Worker Safety and Resilience

| **Section** | **Item** | **Checklist Description** | **Location in Manuscript** |
| --- | --- | --- | --- |
| **TITLE** | 1 | Identify the report as a scoping review. | Title page and manuscript title |
| **ABSTRACT** | 2 | Provide a structured summary that includes background, objectives, methods, results, and conclusions. | Abstract |
| **INTRODUCTION** | 3 | Describe the rationale for the review in the context of what is already known. | Section 1: Introduction |
|  | 4 | State the objectives and/or questions addressed by the review. | Section 1: Introduction, final paragraph |
| **METHODS** | 5 | Specify inclusion criteria used to select sources of evidence. | Section 2.3: Eligibility Criteria |
|  | 6 | Describe information sources and the date range used for the search. | Section 2.1: Search Strategy and Information Sources |
|  | 7 | Present the search strategy, including key terms and databases used. | Section 2.1: Search Strategy and Information Sources |
|  | 8 | State the process for selecting sources of evidence, including screening and eligibility. | Section 2.2: Study Selection and Screening; Figure 1 |
|  | 9 | Describe methods of data charting and extraction, including tools used. | Section 2.4: Data Charting and Extraction |
|  | 10 | Indicate whether critical appraisal of sources of evidence was conducted. | Section 2.5: Synthesis Approach |
|  | 11 | Describe methods for synthesizing results. | Section 2.5: Synthesis Approach |
| **RESULTS** | 12 | Provide the number of sources screened, included, and excluded, with reasons. | Section 2.2: Study Selection and Screening; Section 3: Results; Figure 1; Table S1 |
|  | 13 | Present characteristics of the included sources of evidence. | Tables 1-4 |
|  | 14 | Present results of individual sources of evidence and synthesis. | Sections 3.1-3.5; Tables 1-4 |
| **DISCUSSION** | 15 | Summarize the main results, including key concepts, themes, and evidence gaps. | Section 4: Discussion |
|  | 16 | Discuss limitations of the scoping review process. | Limitations subsection |
|  | 17 | Provide conclusions and implications for policy, practice, and/or research. | Section 4.7: Conclusions |
| **FUNDING** | 18 | Report sources of funding for the review. | Funding statement |
| **OTHER INFORMATION** | 19 | Report any potential conflicts of interest. | Conflicts of Interest section |
|  | 20 | Provide a statement of data availability. | Data Availability Statement |
|  | 21 | Acknowledge contributors and support. | Acknowledgments section |
|  | 22 | Indicate registration or protocol, if applicable. | Section 2.6: Protocol and Registration |

**Confirmation:** This checklist confirms that the essential PRISMA-ScR items have been addressed in the accompanying revised manuscript and supporting information files.
